# Emotional Intelligence and Its Relationship with Levels of Social Anxiety and Stress in Adolescents

**DOI:** 10.3390/ijerph15061073

**Published:** 2018-05-25

**Authors:** Javier Cejudo, Débora Rodrigo-Ruiz, Maria Luz López-Delgado, Lidia Losada

**Affiliations:** 1 Department of Psychology, Faculty of Education of Ciudad Real, University of Castilla La Mancha, Ronda de Calatrava 3, 13071 Ciudad Real, Spain; maluxdjs@hotmail.com; 2 Department of Research Methods and Assessment in Education II, Faculty of Education, UNED, Juan del Rosal, 14, 28040 Madrid, Spain; deborarodrigo@gmail.com (D.R.-R.); llosada@edu.uned.es (L.L.)

**Keywords:** emotional intelligence, adolescence, social anxiety, stress, well-being

## Abstract

The aim of this work is to extend the research on the relationships between emotional intelligence and various indicators of subjective well-being in adolescents, such as stress and social anxiety. The existence of differences in stress and social anxiety as a function of an emotional intelligence score is also analyzed. A total of 505 Spanish adolescents between the ages of 12 and 18 participated in the study. The results obtained support the existence of a positive relationship between trait emotional intelligence and subjective well-being. Likewise, the existence of a negative association between emotional intelligence and stress, and emotional intelligence and social anxiety, is confirmed. These results are consistent with other similar works based on adolescent samples.

## 1. Introduction

Emotional intelligence (EI) has generated considerable interest, as reflected in the different theoretical models, conceptualizations, and assessment measures [1,2,3,4,5,6,7]. In a first approach to the construct, EI has been conceived as “an ability to perceive, assimilate, understand, and regulate emotions in the self and others so as to promote emotional and intellectual growth” [8]. Meanwhile, other researchers [9,10] affirm that EI can be conceived as a constellation of personality traits related to emotion. In particular they define EI as “a constellation of dispositions and self-perceived abilities rather than a class of cognitive-emotional abilities” [9]. These two definitions of the construct have given rise to two streams of research: EI as an ability, consisting of discrete emotional abilities, evaluated through maximum performance tests; and EI as a trait (TEI), consisting of personality dispositions related to emotions, measured by typical performance tests [11].

Usually, typical performance tests (self-reports) are the most used in research because of their ease of application and cost reduction [12]. Specifically, the instruments most frequently used are the Trait Meta-Mood Scale (TMMS) [13] and the Wong and Law Emotional Intelligence Scale (WLEIS) [14], based on the model of Salovey and Mayer [5] and the Trait Emotional Intelligence Questionnaire (TEIQue) [15] from the theoretical approaches of Petrides and Furnham [9]. However, self-reports from EI that come from different theoretical models are not usually used in combination.

One of the most fruitful fields of EI research focuses primarily on providing evidence of the relationship between EI and physical and psychological well-being [16]. As highlighted by Sánchez-Álvarez, Extremera, and Fernández-Berrocal [17], the appropriate use of certain emotional strategies could contribute to experiencing a higher rate of positive emotional states and the reduction of negative emotional states. Therefore, emotional strategies will have a positive impact in the welfare and health of people. In addition, EI could be acting through two complementary processes. On the one hand, EI could reduce the frequency and duration of negative emotions that appear as a consequence of certain stressful events. On the other hand, EI could increase the frequency and maintenance of positive emotions [18]. In this sense, numerous studies show that high scores in EI are linked to the improvement of psychological well-being [19,20,21,22,23]. In addition, several meta-analyses confirm the relationship between TEI and psychological well-being [24,25]. Likewise, other reviews corroborate the relationship between ability EI and well-being [26].

Most of the studies that relate to EI and psychological well-being have been carried out with an adult population. However, studies in the adolescent population are still scarce. Although it is widely assumed that adolescence is a stage of development characterized by a great variety of biological, psychological, and social changes [27,28], these changes experienced by adolescents and their limited experience can turn some vital events into stressors and have negative psychological repercussions. In addition, the accumulation of stressful events can affect psychological well-being and the emotional state. Psychological well-being is influenced not only by the occurrence of stressful events, but also by personal characteristics that determine the assessment of such events and by the way in which the adolescents face the experienced stress [29,30]. These stress factors include the family, school, acceptance of the peer group, the difficulties of early romantic relationships, and uncertainty about the future circumstances of adult life [31].

The research confirms that there are several personal characteristics that are crucial for the adolescent’s psychosocial adjustment [32], including self-esteem [33], social competence [34], and emotional regulation strategies [35,36]. Several studies have already investigated the relationship between EI and psychological functioning, so it is necessary to continue research in this field [3,10,37].

In this regard, Sánchez-Álvarez et al. [17] confirms that positive and negative effects mediate the relationship between EI and satisfaction with life. Balluerka, Gorostiaga, Alonso-Arbiol, and Aritzeta [38] found that EI displayed a positive relationship with adolescents’ well-being. Fernández-Berrocal, Mayor, Extremera, and Pizarro [39] analyzed the relationship between TEI and anxiety, depression, suppression of thoughts, and self-esteem measures in adolescents. The results indicated that TEI correlated negatively with depression and anxiety. On the other hand, Velasco, Fernández, Páez, and Campos [40] found that adolescents belonging to the non-clinical population differed from adolescents with dysphoric characteristics because of their lower scores on emotional attention, anxiety, and suppression of thoughts. Similarly, Zeidner et al. [28] conclude that EI seems to have a moderate influence on emotional functioning during adolescence. Other studies found that EI was associated with the reduced use of maladaptive emotional adjustment strategies and the increasing use of adaptive strategies [41,42].

Among the indicators of psychological well-being most commonly studied, we find the absence of stress and anxiety. However, there are few studies that relate to EI and social anxiety in adolescents. Social anxiety is characterized by constant fear of one or more social or performance situations in which the person is exposed to unknown persons or to the possible scrutiny of others [43]. It is a disorder that hinders the psychosocial adjustment of individuals because of the deep anguish and restlessness it generates [44]. Social anxiety in adolescence is significantly related to other psychopathological disorders, such as loneliness or dysphoria, among others [44], and with difficulties in interpersonal relationships with peers [45,46]. Furthermore, in the educational context, socially anxious students often show avoidance behaviors in response to school work, which makes it difficult to test their learning outcomes [47]. As a result, they may present a lack of socio-school adaptation [48,49]. In addition, adolescents with higher scores in social anxiety present a higher number of victimization behaviors to bullying and cyberbullying [50].

Some studies that address the relationship between TEI and perceived stress have supported the existence of a negative relationship between both variables in adults [51] and university students [52]. Salovey, Stroud, Woolery, and Epel [53] found that high EI among young people between 16 and 23 years old was associated with lower levels of social anxiety and depression, less passive coping, and greater use of active coping strategies, as well as lower levels of rumination and perception of stressors as less threatening. Likewise, some studies confirm that adolescents with lower scores on TEI, specifically in emotional repair, reported a high risk of developing social anxiety characterized by negative thoughts about social interactions [54].

In the present work, different objectives are addressed. First, we intend to extend the previous research regarding the relationship between TEI, stress, and social anxiety in adolescents, given the scarcity of studies in this subgroup. As a second objective, we will analyze the existence of differences in stress and social anxiety as a function of the EI score.

**Figure 1:**
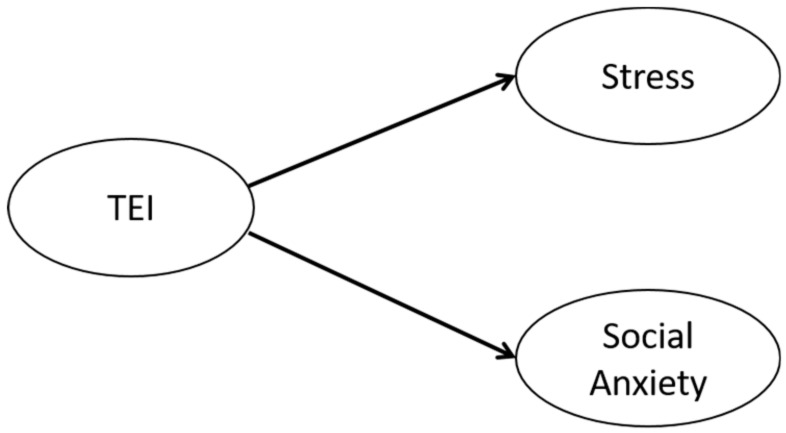
Hypothesized model. TEI—trait emotional intelligence.

According to the model proposed in Figure 1, and based on the findings of previous research, it is expected that (a) stress is negatively predicted by the TEI and (b) the TEI will negatively predict social anxiety.

## 2. Method

### 2.1. Participants

In this explanatory and cross-sectional study, a total of 505 Spanish adolescents from five public education secondary schools participated. The age of the participants ranged from 12 to 18 years (*M* = 14.06, *SD* = 1.89); 55.2% were between 12 and 14 years old, 44.8% were between 15 and 18 years old. In the distribution by gender, 213 (42.18%) were boys and 292 (57.82%) were girls (Table 1). To select the participants, stratified random sampling was established. It should be noted that erroneous questionnaires (*n* = 28) were excluded from the study, as were students who failed to send informed consent (*n* = 18).

### 2.2. Procedure

The collaboration of the Educational Centers of the Province of Toledo (Spain) was requested, based on sampling of the convenience of the categories under study. An authorization model was attached for the adolescents’ legal heads and the anonymity of the collected information was guaranteed, clarifying that its use would be only for scientific purposes. Participants received information about the research objectives and procedure.

### 2.3. Measures

In order to evaluate the variables under study, four assessment instruments were administered with psychometric guarantees of reliability and validity.

Trait Emotional Intelligence Questionnaire Adolescents Short Form (TEIQue-ASF) [4] was used for the evaluation of TEI based on the theoretical model of Petrides and Furnham [9]. The 30 items that make up the TEIQue-ASF questionnaire are scored on a 7-point Likert scale (1 = completely disagree, 7 = completely agree). The general emotional intelligence score (GEI) of the total scale is obtained through the sum of the 30 items of the questionnaire.

Wong and Law’s Emotional Intelligence Scale (WLEIS) [14] was used for the evaluation of TEI based on the theoretical model of Mayer and Salovey [8]. The WLEIS is composed of 16 items scored on a 7-point Likert scale, evaluating the level of emotional intelligence as a personality trait (e.g., “I always know the emotions of my friends through their behaviors”). The measure contains four subscales: appraisal and expression of emotion in the self (SA) (four items), appraisal and recognition of emotion in others (OA) (four items), use of emotions or assimilation (UA) (four items), and regulation of emotions in the self (RE) (four items).

Student Stress Inventory-Stress Manifestations (SSI-SM) [55] consists of 22 items measured on a 5-point Likert scale (not at all, rarely, sometimes, often, and totally) that encompass stress manifestations in three areas: emotional (EM) (10 items), physiological (PM) (6 items), and behavioral (BM) (6 items). The results show a factorial structure of three first-order factors referring to EM, PM, and BM, and a second-order indicative of total stress manifestations (SSI-T).

Social Anxiety Scale for Adolescents (SAS-A) [46]. The SAS-A is composed of 22 items, of which 18 are self-descriptive and the other 4 are distracting elements that are not taken into account for the score. It contains three subscales: (a) fear of negative evaluation (FNE) with eight reagents, (b) anxiety and social avoidance before strangers or new social situations (SAD-N) with six items, and (c) the last subscale includes four reagents that measure anxiety and social avoidance in social situations in general (SAD-G). The response format is Likert-type with five options, from 1 (never) to 5 (always). In addition, a global index of social anxiety (SAS-T) is obtained by adding the scores assigned to each of the items, with the exception of the neutral ones. High scores reflect high levels of social anxiety [46].

### 2.4. Analysis of Results

Initially, reliability coefficients (Cronbach’s alpha), average variance extracted (AVE), and McDonald’s omega coefficient (Ω) were calculated to obtain reliability evidence. In the same way, descriptive statistics and the correlation between the variables were obtained through the Pearson coefficient. Subsequently, to explore possible differences in means as a function of the level of TEI through TEIQue-ASF, two subgroups were created for comparison by means of analysis of variance (ANOVA): a subsample of participants with a low level of EI (students who had obtained scores equal to or lower than the 25th percentile) and a subsample of participants who presented a high level of EI (students who had obtained scores equal to or higher than the 75th percentile). The effect size of these differences was calculated using Cohen’s *d* statistic [56]. Finally, in order to analyze the role played by TEI in the prediction of the level of stress and social anxiety, a structural equation model (SEM) was tested using IBM SPSS Amos version 22 (SPSS Inc., Chicago, IL, USA).

## 3. Results

### 3.1. Descriptive Analysis

Table 2 shows the descriptive statistics and evidence of reliability of each of the instruments used. With respect to reliability, it was confirmed that the internal consistency indices were satisfactory. In relation to Cronbach’s alpha, all the instruments obtained a value higher than 0.70, the minimum value recommended by George and Mallery [57]. With respect to McDonald’s omega, values exceeded the 0.70 minimum value recommended by McDonald [58]. On the other hand, when AVE > 0.50, it can be affirmed that a substantial amount of the variance of the indicators is captured by the construct, compared with that captured by the measurement error. In this sense, in all the scales and subscales, the average variance extracted is higher than 0.50, except for the PM subscale.

Finally, a pattern of relationships as predicted can be seen by observing the correlation matrix, because the relationships between all the variables of TEI and social anxiety and stress are negative (see Table 3).

### 3.2. Predictive Analysis

#### 3.2.1. Significant Differences Depending on the Level of GEI

In order to explore the possible differences of means as a function of the level of GEI (by means of the TEIQue-ASF), two subgroups with high and low GEI were created (as detailed above) for comparison by analysis of variance.

Significant differences were observed in all the variables of social anxiety and stress, except in FM between the group with high and low GEI. The results confirm that students with high GEI have lower scores in social anxiety and stress than students with low GEI.

In order to assess the magnitude of these differences, the effect size for each variable was calculated (see Table 4).

#### 3.2.2. Structural Equation Model

To test the hypothesized global model, an analysis of structural equations was used with the maximum likelihood procedure that—although it assumes multivariate normality—is reasonably robust to its non-compliance [59].

According to the approaches of the structural equation model approach, in the same model we include the constructs of TEI, social anxiety, and stress, determining the weights in the relationships that occur between them, using the indications of Schreiber, Nora, Stage, Barlow, and King [60] and Schweizer [61] for their interpretation.

**Figure 2:**
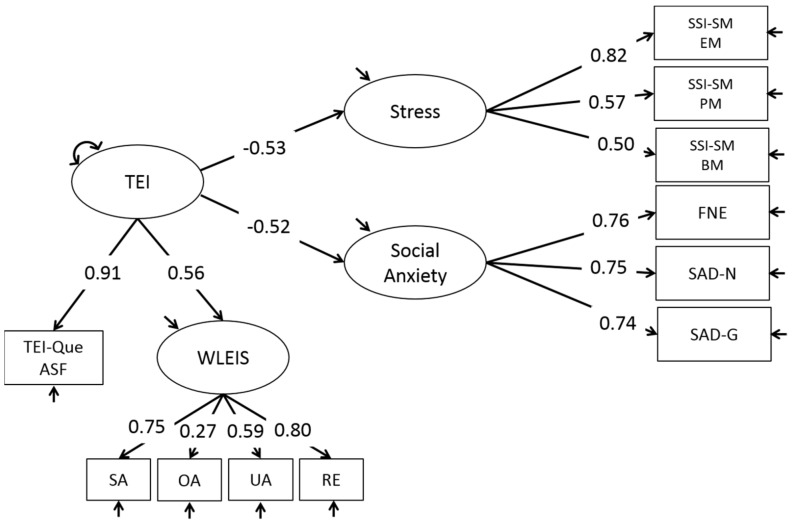
Structural equations model with standardized estimated parameters for the variables TEI, social anxiety, and stress. TEI = Trait Emotional Intelligence; TEIQue-ASF = Trait Emotional Intelligence Questionnaire Adolescents Short Form; WLEIS = Wong and Law´s Emotional Intelligence Scale; SA = appraisal and expression of emotion in the self; OA = appraisal and recognition of emotion in others; UA = use of emotions or assimilation; RE = regulation of emotions in the self; SSI-SM = Student Stress Inventory-Stress Manifestations; EM = Emotional Manifestations; PM = Physiological Manifestations; BM = Behavioral Manifestations; FNE = Fear of Negative Evaluation; SAD-N = Social Avoidance Distress-New; SAD-G = General Social Avoidance and Distress.

In our model, we present the latent variable exogenous TEI measured through the TEI-Que ASF and WLEIS tests, which extracts the variance shared by the variables social anxiety and stress (see Figure 2).

The model presents a very good fit: χ^2^ = 58.57 (*df* = 41). χ^2^/dL = 1.43, TLI = 0.95, CFI = 0.97, RMSEA = 0.04; probability level (0.037). As noted, all the indicators are within the appropriate limits; χ^2^/dL is less than 2, TLI is not less than 0.95, the CFI indicator is above 0.90, and RMSEA is less than 0.06. In short, we have a model with a good fit. Additionally, the standardized estimates offered in Figure 2 follow the predicted direction with negative effects of TEI on stress and social anxiety, thus it should be noted that, in effect, TEI predicts the constructs of social anxiety and stress in the adolescent population. In both cases, it is a negative prediction. As observed in Figure 2, the weights obtained over TEI from the latent variables, social anxiety and stress, are quite similar; thus we affirm that the weight of TEI on social anxiety and stress is very similar. On the other hand, among the different manifestations measured for stress, it is the emotional manifestations that have the greatest weight in the model; substantially greater than the physiological and behavioural manifestations. On the other hand, we did not observe differences in relevance between the different factors measured in the latent social anxiety variable. Another observation is the difference in the weights obtained for the different TEI instruments. The results are very favourable for the TEI-Que ASF, whose weight is higher than that calculated for the WLEIS. Finally, among the different dimensions of the WLEIS, emotional regulation has the highest weight contributing to the latent variable TEI.

## 4. Discussion

The main objective of this work was to extend previous research on the relationships between EI and some indicators of subjective well-being in adolescents, namely stress and social anxiety. The results obtained support the existence of a positive relationship between TEI and subjective well-being; specified as the existence of a negative association between EI and stress, and between EI and social anxiety. These results are consistent with other similar work with adolescents [17,28,38,39,40,53,62].

It is likely that high EI is associated with high well-being when the experience of stress and social anxiety is lower, in coherence with the postulates of Schiffrin and Nelson [63] of an inverse relationship between happiness and stress. These findings would support the idea that EI may be associated with more effective confrontation with stress, promoted by a greater emotional understanding of oneself and others and, therefore, a better prediction of the results of coping attempts. On the other hand, EI can be associated with superior social functioning, through greater emotional understanding of others and more adaptive interpersonal relationship skills, thus fostering more pleasant social interactions. Furthermore, these results support the idea that EI could act by reducing the frequency and duration of negative emotions that appear as a consequence of certain stressful events and social interactions [64].

However, the results of the emotional intelligence variable assessed by the TEIque-ASF indicate a stronger relationship with social anxiety than with stress, while in the different variables of emotional intelligence evaluated through the WLEIS, the relationships are similar. In this sense, the results obtained are in the same direction as other previous studies that have shown high negative relationships between EI and the trait of neuroticism belonging to the Big Five personality theory [65,66].

The second objective of the present study was to analyze the existence of differences in stress and social anxiety as a function of the EI score, in line with the results obtained in previous studies [54]. In this sense, the results obtained are partially congruent with those found by Castella et al. [54], because adolescents with lower levels of emotional regulation have more symptoms of stress and social anxiety. However, in the work of Castella et al. [54], no differences were found in emotional clarity and emotional attention depending on the level of EI; whereas in the present study, adolescents with higher EI scores have lower levels of stress and social anxiety. A possible explanation for these results may the high relation, in comparison with other self-reports, presented by TEI, evaluated through the TEI-Que ASF, with variables related to mental health [24,67].

On the other hand, there is a difference in weights obtained by the different instruments of emotional intelligence in their contribution to the latent variable TEI. The results have indicated higher weights for TEI-Que ASF, in relation to WLEIS. The information collected for TEI comes from a greater part of the TEI-Que ASF, and less from the measures of social anxiety and stress of the students. Likewise, within the dimensions of WLEIS, emotional regulation stands out for its greater contribution to the TEI variable. One possible explanation for these results may be the role that emotional regulation plays in adapting people to the demands of the social environment. Emotional regulation strategies can help adolescents overcome stressful situations and allow them to improve their interpersonal relationships and grow emotionally.

There are multiple reasons why the contributions of this study are limited, such as the fact that participants have not been selected by random procedures. To this must be added the fact that the data have been obtained by self-report, which becomes a common source of variance that affects the results. In addition, self-reports may be influenced by concerns of social desirability that adolescents may have. Finally, a limitation of the study could be the size of the sample used. Future studies should be carried out with much larger sample sizes to allow for better generalizability in the adolescent population. Because the data shows a nested structure (students in classes), we also point out this aspect as a limitation of the study.

In order to suggest improvements to be taken into account for future research, it is suggested that a longitudinal design would allow for proposing truly causal models in which the influences of identification on behaviors over time are appreciated. It would also be opportune to have a wider range of measures that allow us to test a more complex model.

Thus, this study presents novel contributions with respect to other previous work: it jointly analyzes two measures of EI as a trait, thus allowing the comparison between both measures and their relation with stress and social anxiety, the variables scarcely studied in the adolescent group. In addition, it analyzes the incidence of the level of EI in stress and social anxiety in adolescents, opening a debate on the role it plays in the prevention of both. It can also help professionals in psychology and psychotherapy in general to improve the treatment of adolescents with pathological stress and social anxiety.

## 5. Conclusions

In conclusion, these results indicate that EI as a personality trait has predictive power over social anxiety and stress in a sample of adolescents. The current study could provide valuable information for the design, implementation, and evaluation of programs for the development of EI, for the promotion, prevention, and intervention of emotional problems in adolescence. EI programs could promote an improvement of one’s own emotional comprehension and of others, as well an optimization of intra- and interpersonal emotional regulation processes in adolescence.

## Figures and Tables

**Table 1:** Sociodemographic data. Sex, grade, and residential area of the sample.

		*n*	%
Sex	Male	213	42.18%
	Female	292	57.82%
Grade	1st Grade	157	31.09%
	2nd Grade	125	24.75%
	3rd Grade	117	23.17%
	4th Grade	106	20.99%
Residential area	Urban *	174	34.46%
	Rural **	331	65.54%

Note: * >10,000 inhabitants; ** <10,000 inhabitants.

**Table 2:** Descriptive statistics of the variables under study and reliability evidence of the instruments used (*n* = 505). AVE—average variance extracted; *SD*—standard deviation.

	*Mean*	*SD*	α	AVE	Ω
**TEIQue-ASF**					
GEI	4.69	0.60	0.87	0.623	0.931
**WLEIS**					
SA	20.55	4.64	0.72	0.501	0.716
OA	20.98	4.04	0.73	0.523	0.724
UA	20.10	4.18	0.70	0.506	0.701
RE	18.09	5.31	0.78	0.502	0.789
**SSI-SM**					
SSI-T	44.22	10.19	0.81	0.579	0.893
EM	21.66	6.30	0.81	0.565	0.809
PM	11.16	3.34	0.70	0.489	0.723
BM	11.40	3.18	0.72	0.523	0.701
**SAS-A**					
SAS-T	2.54	0.71	0.89	0.588	0.893
FNE	2.56	0.79	0.80	0.506	0.815
SAD-N	2.76	0.85	0.77	0.512	0.734
SAD-G	2.20	0.89	0.78	0.489	0.756

Notes: TEIQue-ASF = Trait Emotional Intelligence Questionnaire Adolescents Short Form; GEI = General Emotional Intelligence; WLEIS = Wong and Law´s Emotional Intelligence Scale; SA = appraisal and expression of emotion in the self; OA = appraisal and recognition of emotion in others ; UA = use of emotions or assimilation; RE = regulation of emotions in the self; SSI-SM = Student Stress Inventory-Stress Manifestations; SSI-T = Total Student Stress Inventory; EM = Emotional Manifestations; PM = Physiological Manifestations; BM = Behavioral Manifestations; SAS-A = Social Anxiety Scale for Adolescents; SAS-T = Total Social Anxiety Scale; FNE = Fear of Negative Evaluation; SAD-N = Social Avoidance Distress-New; SAD-G = General Social Avoidance and Distress.

**Table 3:** Pearson correlations between variables under study.

	GEI	SA	OA	UA	RE
SSI-T	−0.35 **	−0.18 **	−0.18 *	−0.26 **	−0.19 **
EM	−0.37 **	−0.16 **	−0.19 *	−0.29 **	−0.18 **
PM	−0.13	−0.14	−0.11	−0.12	−0.09
BM	−0.27 **	−0.12	−0.08	−0.14	−0.17 *
SAS-T	−0.42 **	−0.13	−0.34	−0.18 *	−0.19 **
FNE	−0.36 **	−0.16 *	−0.31	−0.19 **	−0.20 **
SAD-N	−0.31 **	−0.06	−0.02	−0.09	−0.13 **
SAD-G	−0.36 **	−0.08	−0.01	−0.17 **	−0.12

Notes: GEI = General Emotional Intelligence; SA = appraisal and expression of emotion in the self; OA = appraisal and recognition of emotion in others; UA = use of emotions or assimilation; RE = regulation of emotions in the self; SSI-T = Total Student Stress Inventory; EM = Emotional Manifestations; PM = Physiological Manifestations; BM = Behavioral Manifestations; SAS-T = Total Social Anxiety Scale; FNE = Fear of Negative Evaluation; SAD-N = Social Avoidance Distress-New; SAD-G = General Social Avoidance and Distress; * *p* < 0.05; ** *p* < 0.01.

**Table 4:** Mean, standard deviation (*SD*), analysis of variance (ANOVA), and size of the effect of the differences in means (Cohen’s *d*) as a function of GEI.

	Low GEI (*n* = 61)		High GEI (*n* = 62)					Levene’s Test	
Variables	*Mean*	*SD*	*Mean*	*SD*	*F*	*p*	*d*	*F*	*p*
SSI-T	48.22	10.94	39.67	9.98	13.852	0.000	0.82	0.903	0.345
EM	23.93	6.93	18.53	5.71	15.080	0.000	0.85	3.386	0.307
PM	11.41	3.77	10.50	3.04	1.485	0.226	0.27	1.056	0.307
BM	12.88	3.66	10.64	2.98	9.328	0.003	0.67	2.953	0.090
SAS-T	2.88	0.72	2.19	0.60	28.698	0.000	1.04	2.399	0.124
FNE	2.88	0.86	2.24	0.72	17.147	0.000	0.81	0.901	0.345
SAD-N	3.08	0.80	2.43	0.78	17.943	0.000	0.82	0.073	0.787
SAD-G	2.56	0.95	1.79	0.68	23.221	0.000	0.93	5.570	0.062

Notes: GEI = General Emotional Intelligence; SSI-T = Total Student Stress Inventory; EM = Emotional Manifestations; PM = Physiological Manifestations; BM = Behavioral Manifestations; SAS-T = Total Social Anxiety Scale; FNE = Fear of Negative Evaluation; SAD-N = Social Avoidance Distress-New; SAD-G = General Social Avoidance and Distress; Cohen’s *d* index for the effect size of the differences of each variable.
